# Quality Evaluation of the Root Bark Epidermis of Peony by HPLC-DAD-ESI-MS/MS

**DOI:** 10.3390/molecules31040588

**Published:** 2026-02-08

**Authors:** Huimin Xiao, Xinwen Huang, Feiyu Xie, Mengzhen Fan, Yanhua Xie, Siwang Wang, Jinming Gao

**Affiliations:** 1 Shaanxi Key Laboratory of Natural Products & Chemical Biology, College of Chemistry & Pharmacy, Northwest A&F University, Yangling 712100, China; xiaohuimin_99@163.com; 2 Shaanxi Fengdan Zhengyuan Biotechnology Limited Company, Xi’an 710076, China; 3 Department of Life Science and Medicine, Northwest University, Xi’an 710068, China; 14770819466@163.com (X.H.); m15667206513@163.com (F.X.); fanmengzhen@stumail.nwu.edu.cn (M.F.); xieyanh@fmmu.edu.cn (Y.X.); wangsiw@fmmu.edu.cn (S.W.)

**Keywords:** peony, epidermis, high performance liquid chromatography, fingerprint, mass spectrometry, active components

## Abstract

The annual output of Moutan Cortex is significant, but the epidermis of the root bark of peony (EPRP), as a by-product of Moutan Cortex, is typically discarded. To achieve full use of resources, this study aimed to develop an HPLC fingerprint analysis method using HPLC-DAD-ESI-MS/MS on EPRP sourced from different regions to establish EPRP quality control standards. For chromatography, a ShimPack Scepter C_18_ column (4.6 mm × 250 mm, 5 μm) was used. The mobile phase for HPLC consisted of acetonitrile (A) and a 0.1% aqueous formic acid solution (B). An HPLC fingerprint was established, featuring 30 characteristic peaks with a similarity of over 0.80. A total of 31 components were identified, with 22 chemical markers determined, including 1-galloylglucose, gallic acid, methyl gallate, oxypaeoniflora, paeonolide, apiopaeonoside, albiflorin, paeoniflorin, *p*-coumaric acid, ferulic acid, 1,2,3,6-tetragalloylglucose, ellagic acid, galloylpaeoniflorin, luteoloside, 1,2,3,4,6-O-penta-galloylglucose, diosmin, neodiosmin, resveratrol, mudanpioside C, benzoyloxypaeoniflorin, benzoylpaeoniflorin, and paeonol. These markers align with component structure theory, allowing for the analysis of the structural characteristics of EPRP from different regions. These findings provide a valuable reference for the future quality evaluation of EPRP, enhance the understanding of the components in EPRP from diverse sources, and lay a foundation for the development and greater utilization of EPRP.

## 1. Introduction

**Figure 1:**
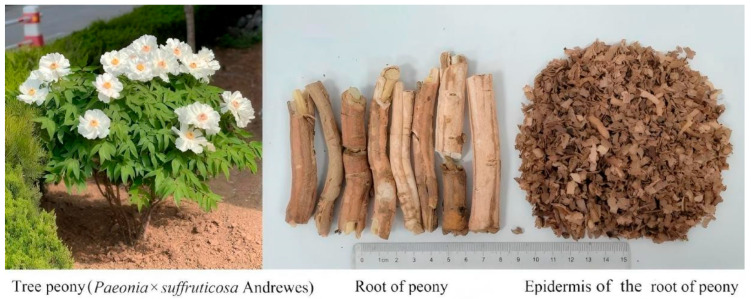
Tree peony, root of peony, and epidermis of the root of peony (EPRP).

Moutan Cortex is the dried root bark of the peony plant (*Paeonia suffruticosa* Andr., Figure 1), a member of the Ranunculaceae family. Besides Moutan Cortex, the leaves, flowers, pollen and seeds of peony are also utilized, while the epidermis of the root bark of peony (EPRP), the woody cores, and the fruit pods are discarded. Volume 1 of the Chinese Pharmacopoeia (2025 edition) includes Moutan Cortex (the root bark of peony), and states that the effects of Moutan Cortex include clearing heat, cooling blood, and promoting blood circulation and resolving stasis [1]. 2022 edition of the Shandong Medicinal Materials Standard and the 2023 edition of the Henan Medicinal Materials Standard include peony leaves, and states that their effects include detoxification and stopping dysentery. 1980 edition of the Gansu Traditional Chinese Medicine Processing Specifications include peony flowers and states that their effects include clearing heat and cooling blood. “Shen Nong’s Herbal Classic” and “Compendium of Materia Medica” include peony pollen and states that their effects include promoting blood circulation, regulating menstruation, clearing heat and detoxifying. The peony seeds are pressed for oil as a food product.The EPRP is the epidermis of the dried root bark of the peony plant, a member of the Ranunculaceae family. When the root is harvested in autumn, the woody core is removed, fine roots and sand are removed, and the epidermis of the roots is scraped off. The epidermis is washed and dried, and the resulting product is referred to as the epidermis of the root bark of peony (also known as “scraped bark”, or EPRP).

EPRP is a processing by-product of Moutan Cortex. The peony planting area in China exceeds 60,000 hectares, with an annual output of over 1000 tons. Moutan Cortex is mainly distributed in Anhui, Shaanxi, Shandong, Henan, Sichuan, and Chongqing, and a large amount of EPRP is produced. Current research shows that Moutan Cortex is rich in many compounds, including terpenoids (oxypaeoniflorin, albiflorin, paeoniflorin, galloylpaeoniflorin, mudanpioside C, benzoyloxypaeoniflorin, benzoylpaeoniflorin), phenolic and phenolic glycosides (paeonolide, apiopaeonoside, paeonol), tannic acids (gallic acid, catechin, methyl gallate, benzoic acid, 1,2,3,6-tetra-O-galloyl-D-glucose, 1,2,3,4,6-pentagalloylglucose), and flavonoids (luteoloside, diosmin, neodiosmin). These compounds exhibit anti-inflammatory, anti-coagulation, anti-fibrosis, anti-tumor, liver-protective, and lipid metabolism regulatory effects [2,3,4,5,6,7].

The application of a single or a few quality indicators in a quality assessment of a traditional Chinese medicine (TCM) material is insufficient to comprehensively reflect its overall quality. Because the effective components of TCM are often part of a complex multicomponent system, their therapeutic effects may be related to the combined action of multiple components. Therefore, relying on a single high-content ingredient to evaluate quality cannot accurately represent the therapeutic efficacy of the material. Accordingly, researchers are increasingly using multi-component indices to evaluate the overall quality of TCM [8,9,10,11,12]. For example, many researchers have proposed a component structure theory and fingerprint analysis to qualitatively evaluate TCM treatments based on an overall view, which offers a novel approach for future research into TCM quality control [2,4,13,14,15,16]. A fingerprint analysis of TCM herbal medicines is an effective means of providing overall quality assessment and control of Chinese medicine by reflecting the complex relationships among its chemical components [2]. For the purposes of the present study, individual EPRP samples with high homogeneity were grouped into categories based on fingerprints, distinguishing them according to their authenticity, origin, and processing methods and the quality of their TCM preparations. Principal component analysis (PCA) [17] is a data evaluation method based on projection in which an unsupervised model generates a number of new variables by linearly combining the multidimensional variables of the original data matrix with a certain weight. This identification method can simply and intuitively reflect the differences between samples and is a commonly used fingerprint analysis method in TCM. Partial least squares-discriminant analysis (PLS-DA) [18,19] is a statistical analysis method that integrates PCA, canonical correlation analysis, and multiple linear regression analysis. As a supervised pattern recognition method based on partial least squares, it has a high prediction accuracy and wide applicability. Moreover, it offers certain advantages when used to comprehensively analyze multidimensional information related to the quality of TCM.

Currently, a UPLC-Q-TOF-MS method was applied to analyz 10 main components in EPRP [8]. There are no Chinese Materia Medica standards for EPRP or systematic studies. EPRP has been speculated to have the same chemical components and similar pharmacological effects as Moutan Cortex. Therefore, in the context of our ongoing exploration of components from TCMs [20,21], the present study established an HPLC fingerprint pattern, with 31 chemical components identified by comparing with reference materials and by HPLC-DAD-ESI-MS/MS, and with 22 chemical markers determined for EPRP that had been sourced from different regions. Guided by the component structure theory and HPLC fingerprinting, we explored the unique composition of EPRP from different regions to provide a valuable reference for future Chinese Materia Medica quality evaluations, and this study fills the gap of no quality control methods for EPRP.

## 2. Results and Discussion

### 2.1. Optimum Conditions for HPLC Analysis

The HPLC analysis was optimized by comparing various elution systems, including acetonitrile with 0, 0.05%, 0.1%, 0.2%, and 0.3% formic acid in water and methanol with 0, 0.05%, 0.1%, 0.2%, 0.3% formic acid. The results indicated that using acetonitrile with 0.1% formic acid as the mobile phase provided the best separation and peak shape. Additionally, extraction solvents (methanol, 50% methanol in water, 70% methanol in water and alcohol, 50% alcohol in water, 70% alcohol in water), the ultrasonic extraction time (20 min, 30 min, 40 min, 45 min, and 60 min), detection wavelength (200–400 nm), column temperature (20 °C, 25 °C, 30 °C, and 35 °C), and flow rate (0.5, 0.8, 1.0, and 1.2 mL·min^−1^) were examined. Ultimately, a 70% methanol–water ultrasonic extraction for 45 min, with 270 nm as the detection wavelength and 1.0 mL·min^−1^ as the flow rate, was selected. Under these conditions, there was a relatively ideal peak shape (the tailing factor ranges from 0.95 to 1.05) and degree of separation (the separation degree is not less than 1.2).

### 2.2. Establishing an HPLC Fingerprint of EPRP

#### 2.2.1. Method Validation of the Fingerprints

To obtain a repeatable and stable chromatographic fingerprint of EPRP for quality control, the precision, reproducibility, and stability of the method were determined by the relative standard deviation (RSD) value of the average relative retention times (RRTs) and relative peak areas (RPAs) of the 30 common characteristic peaks, using peak 29 as the reference. The precision of the method was obtained by successively analyzing the same sample solution six times. The results demonstrated that the RSDs of the relative peak areas when evaluating precision did not exceed 0.26% for the RRTs and 3.06% for the RPAs. Reproducibility was evaluated with six independently prepared sample solutions. The results demonstrated that the variation in the RRTs and RPAs of the characteristic peaks did not exceed 0.32% and 3.66%, respectively. Stability testing was performed by analyzing the same sample solution at different times (0, 2, 4, 8, 12, and 24 h). The results revealed that the RSDs of the relative peak areas when evaluating stability were below 0.28% for the RRTs and 3.17% for the RPAs. These results confirmed that the HPLC fingerprint analysis method was both valid and satisfactory (Table 1).

#### 2.2.2. Establishing a Characteristic HPLC Fingerprint

**Figure 2:**
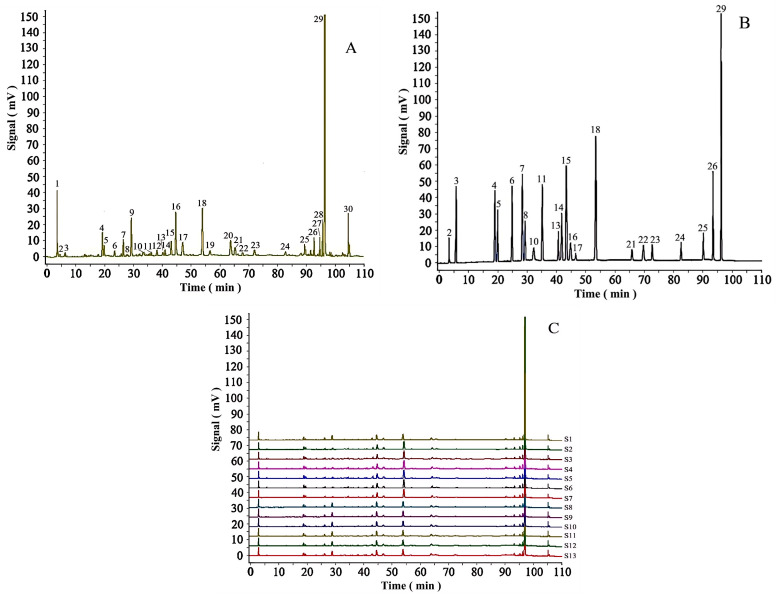
(**A**) Characteristic spectrogram of the chromatographic fingerprint. Peaks 1–30 are characteristic peaks. (**B**) Mixed reference substances. Components: 2, 1-galloyl glucose; 3, gallic acid; 4, methyl gallate; 5, oxypaeoniflora; 6, paeonolide; 7, apiopaeonoside; 8, albiflorin; 10, paeoniflorin; 11, *p*-coumaric acid; 13, ferulic acid; 14, 1,2,3,6-tetragalloylglucose; 15, ellagic acid; 16, galloylpaeoniflorin; 17, luteoloside; 18, 1,2,3,4,6-O-penta-galloylglucose; 21, diosmin; 22, neodiosmin; 23, resveratrol; 24, mudanpioside C; 25, benzoyloxypaeoniflorin; 26, benzoylpaeoniflorin; and 29, paeonol (reference peak). (**C**) Chromatographic fingerprints and characteristic peaks of the 13 batches of EPRP (samples 1–13 labeled “S1,” “S2,” …).

Thirteen batches of EPRP samples were analyzed using HPLC to establish a characteristic and comprehensive fingerprint, with standardization performed using software from the National Pharmacopoeia Committee. A total of 30 common peaks were identified in the HPLC fingerprint, which formed the reference chromatogram. By superimposing the fingerprints of each batch, sample fingerprints for the 13 batches of EPRP were ultimately obtained (Figure 2).

Of the 30 characteristic peaks observed, peak 29 was chosen as the reference peak due to its high content, high intensity, and good degree of separation in the EPRP chromatograms. RRTs and RPAs with respect to the reference peak were then measured and included in the statistics of the HPLC fingerprints. The results showed that the RSDs of the RRTs of the 30 characteristic peaks in the 13 batches of EPRP samples were less than 1.0%, whereas the RSDs of the RPAs of the common peaks were less than 10%. These results indicated that the RRTs among the characteristic peaks were relatively stable. However, the RSDs of the RPAs were quite different, indicating a difference in the content of each component in the EPRP obtained from different regions.

The similarities, calculated by comparing the complete chromatographic profiles of the 13 batches of EPRP and the reference chromatogram, were obtained using the Similarity Evaluation System for the Chromatographic Fingerprint of Traditional Chinese Medicine software, as recommended by the former State Food and Drug Administration (now the National Medical Products Administration). The similarity values of the 13 batches of samples S1–S13 were, respectively, 0.976, 0.979, 0.96, 0.912, 0.906, 0.825, 0.824, 0.925, 0.928, 0.870, 0.863, 0.928, and 0.932, indicating that the chemical compositions of the EPRP samples from different regions were similar.

### 2.3. Stoichiometric Analysis

#### 2.3.1. CA

**Figure 3:**
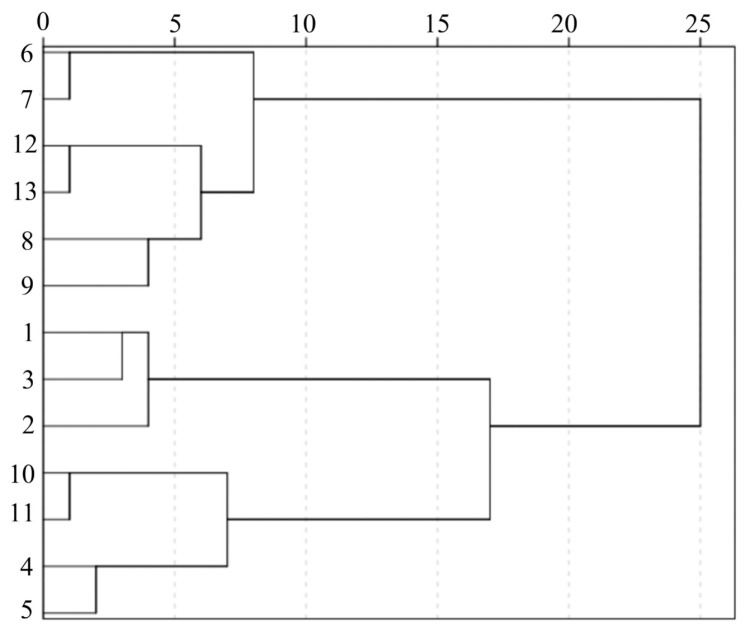
Dendrogram plotting the clustering results of the fingerprints of the 13 batches of EPRP.

Cluster analysis (CA) was carried out using SPSS (version 21.0) software (IBM, Armonk, NY, USA) to calculate similarity measures through Euclidean distances. The results are presented in a dendrogram (Figure 3), in which the degree of association among samples depends upon distance; the shortest distances indicate the highest degree of relationship, and consequently, those objects with those attributes are considered to be the same group. As shown in Figure 3, the 13 batches corresponding to the different regional samples of the EPRP were grouped into three main clusters. The first cluster (cluster I) included samples from batches S6, S7, S8, S9, S12, and S13; the second cluster (cluster II) consisted of samples from batches S1, S2, and S3; and the third cluster (cluster III) consisted of samples from batches S4, S5, S10, and S11. In addition, within cluster I, batches S6 and S7 showed a smaller distance and were defined as one class; S8, S9, S12, and S13 formed another group. These results suggested that the samples of EPRP from distinct regions exhibited certain differences.

#### 2.3.2. PCA and PLS-DA

**Figure 4:**
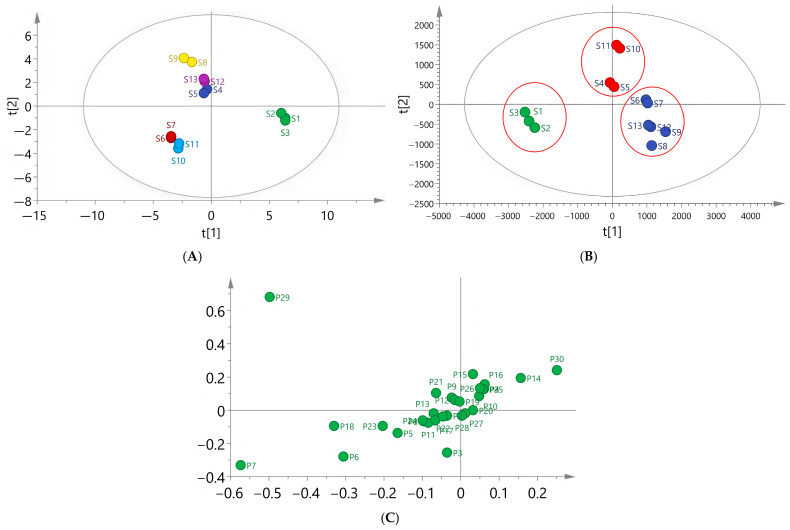
(**A**) PCA score plot of the 13 samples. (**B**) PLS-DA score plot of the 13 samples. (**C**) PLS-DA loading plot of the 30 characteristic peaks.

The peak area of the 30 common peaks of the 13 batches of EPRP samples from different regions was imported as a variable into SIMCA (version 14.1) multivariate statistical analysis software for PCA (Sartorius Stedim Data Analytics AB, Umeå, Sweden). The results showed that the four principal components with the largest contribution described 93.2% of the variability among the samples, indicating that there were significant differences between the EPRP sample groups from different regions (Figure 4A). To ascertain the inter-group differences and component differences among the EPRP samples from different regions, a PLS-DA model was established. The four principal components were extracted. The model quality parameter R2X was 0.934, R2Y was 0.985, and the prediction ability parameter Q2 was 0.873, all of which were higher than 0.5, indicating that the established model had strong interpretation and prediction rates.

As shown in the PLS-DA score plot (Figure 4B), the EPRP from Heyang (batches S1–S3) were grouped together, the EPRP samples from Chengdu (batches S4 and S5) and Dianjiang (batches S10 and S11) were grouped together, and the EPRP samples from Tongling (batches S6 and S7), Heze (batches S8 and S9), and Luoyang (batches S12 and S13) were grouped together. This result was basically consistent with the CA results. According to the results shown in Figure 4C, peaks 3, 5, 6, 7, 14, 18, 23, 29, and 30 may serve to distinguish the EPRP samples from different regions.

### 2.4. Identification of the Chemical Composition

#### 2.4.1. Mixed Reference Standard Identification

**Figure 5:**
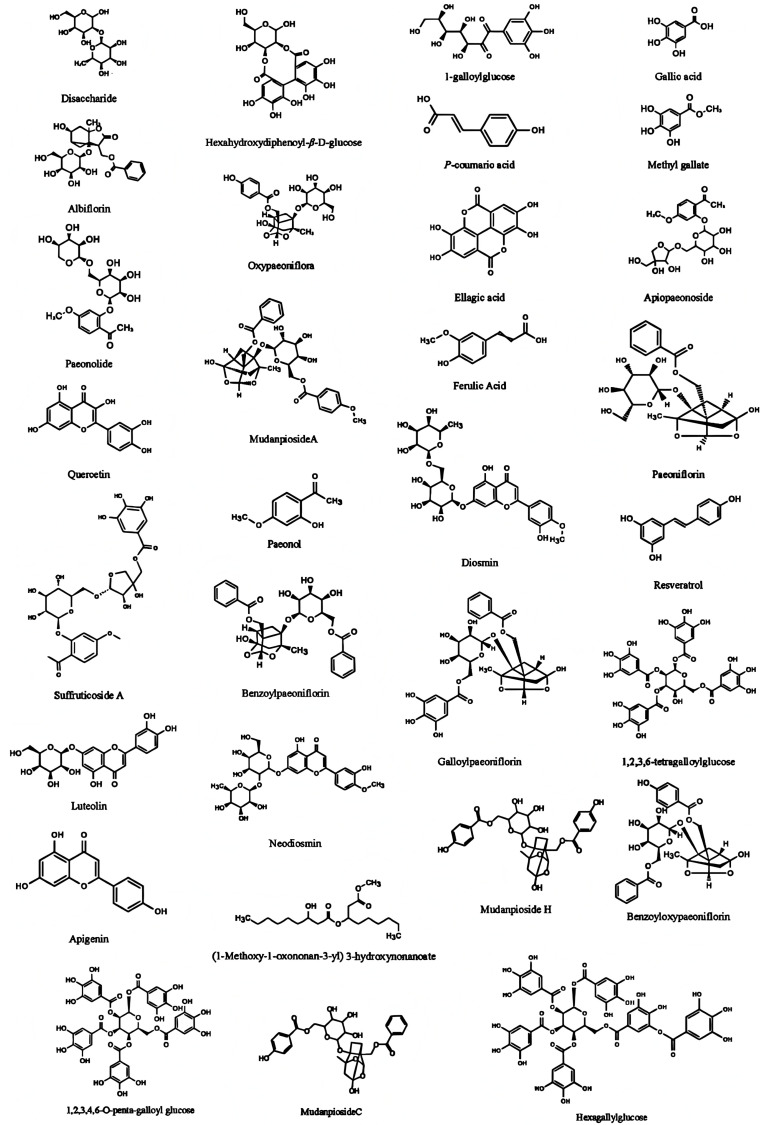
Structures of the 31 compounds.

Based on the analysis described in Section 2.2.2, chromatographic peaks of the test sample, the mixed reference standard, and the blank solvent were compared. The peaks were identified as follows: peak 2, 1-galloylglucose; peak 3, gallic acid; peak 4, methyl gallate; peak 5, oxypaeoniflora; peak 6, paeonolide; peak 7, apiopaeonoside; peak 8, albiflorin; peak 10, paeoniflorin; peak 11, p-coumaric acid; peak 13, ferulic acid; peak 14, 1,2,3,6-tetragalloylglucose; peak 15, ellagic acid; peak 16, galloylpaeoniflorin; peak 17, luteoloside; peak 18, 1,2,3,4,6-O-penta-galloylglucose; peak 21, diosmin; peak 22, neodiosmin; peak 23, resveratrol; peak 24, mudanpioside C; peak 25, benzoyloxypaeoniflorin; peak 26, benzoylpaeoniflorin; and peak 29, paeonol. The results are shown in Figure 2A,B and Figure 5.

#### 2.4.2. Identification by LC-MS/MS

**Figure 6:**
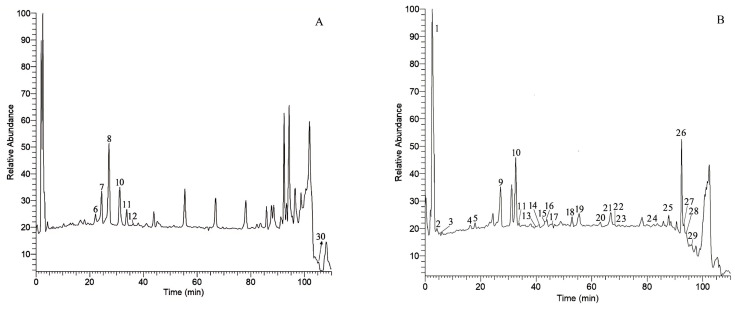
Total ion chromatogram of EPRP under different ion modes. (**A**) Positive ion mode. (**B**) Negative ion mode.

Through a literature comparison and high-resolution mass spectrometry data analysis, 30 feature peaks were identified. The results are shown in Figure 5 and Figure 6 and Table 2. Figure 5 shows the structural formulas of the 31 components, Figure 6 shows the positive and negative ion scanning results of EPRP (From S1, 17 April 2025, Heyang, Shaanxi, China), and Table 2 presents the HPLC-MS/MS-based identification of EPRP. According to the results shown in Table 2, (1-methoxy-1-oxononan-3-yl) 3-hydroxynonanoate was first identified in EPRP, and the characteristic fingerprint of peak 1 contained two compounds (disaccharide and hexahydroxydiphenoyl-β-D-glucose).

### 2.5. Content Determination of the 22 Components

#### 2.5.1. Validation of the Content Determination Method

Table 3 presents the results of the validation of the study’s methodology. All calibration curves showed good linearity (R^2^ > 0.9990) within the determination range. Satisfactory sensitivity was found for all analytes. The RSDs (%) of the interday, intraday, stability, and repeatability tests of the 22 analytes were 0.52–1.85%, 0.46–1.78%, 0.58–1.94%, and 0.64–2.31%, respectively. The average recovery rate was 97.01–100.57%, with an RSD of 1.09–2.39%. These results confirmed the reliability of the developed method.

#### 2.5.2. Analysis of the Chemical Composition

We screened and characterized the components of the EPRP, including terpene nucleosides (oxypaeoniflorin, albiflorin, paeoniflorin, galloylpaeoniflorin, mudanpioside C, benzoyloxypaeoniflorin, benzoylpaeoniflorin), phenolic and phenolic glycoside components (paeonolide, apiopaeonoside, p-coumaric acid, ferulic acid, resveratrol, paeonol), tannic acids (1-galloylglucose, gallic acid, methyl gallate; 1,2,3,6-tetra-O-galloyl-D-glucose, ellagic acid, 1,2,3,4,6-pentagalloylglucose), and flavonoids (luteoloside, diosmin, neodiosmin). The contents of these 22 characteristic compounds in EPRP were determined, with the results summarized in Table 4.

**Figure 7:**
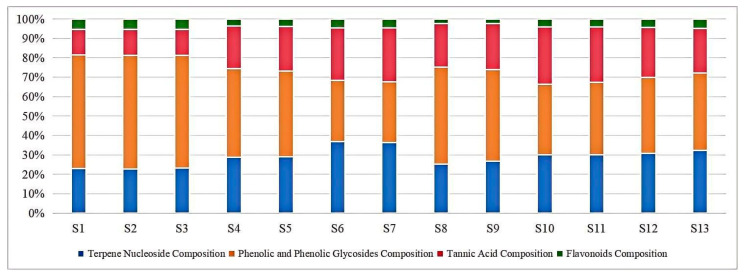
Component percentages of the 13 batches of EPRP.

The mean content of four of the components in the 13 batches of EPRP samples was close to the median, and therefore, the mean content of the four components was used to represent the quantity and quantity ratios of the terpene nucleoside, phenolic and phenolic glycoside, tannic acid, and flavonoid composition of the EPRP sample. The quantity of these four components and their ratio in batches S1–S3 were 22.63, 49.30, 11.29, and 2.06 mg·g^−1^ and 1.00:2.18:0.50:0.09, respectively; in S4, S5, S10, and S11, they were 34.95, 40.81, 26.58, and 1.98 mg/g and 1.00:1.17:0.76: 0.06, respectively; and in S6–S9, S12, and S13, they were 30.03, 30.58, 20.27, and 1.57 mg·g^−1^ and 1.00:1.02:0.67:0.05, respectively. The relationship between the ratio of the components in batches S4, S5, S10, and S11 was closer to that of batches S6–S9, S12, and S13, but there was a difference between the quantities of the components. The relationship between the quantity and quantity ratio of the components in batches S1–S3 was quite different from those in batches S4–S13. This result was consistent with the results of the PLS-DA, indicating that these 22 characteristic compounds represent important differences between the EPRP samples from different regions. Batches S1–S3 were from Shaanxi Heyang Peony Organic Plantation, while other batches were from ordinary cultivated peonies in different regions within China. Our results suggest that divergent planting conditions corresponded to the differences between the EPRP samples. The relative content percentages of the 13 batches of EPRP samples are shown in Figure 7.

## 3. Materials and Methods

### 3.1. Plant Materials, Reagents, and Instruments

The reference substances, including 1-galloylglucose (serial number: G922700, purity ≥ 98%), gallic acid (serial number: HG230820, purity ≥ 98%), methyl gallate (serial number: HA063031, purity ≥ 98%), oxypaeoniflorin (serial number: HO003023, purity ≥ 98%), paeonolide (serial number: HM188951, purity ≥ 98%), apiopaeonoside (serial number: HA185892, purity ≥ 98%), albiflorin (serial number: HA003021, purity ≥ 98%), paeoniflorin (serial number: HP003024, purity ≥ 98%), *p*-coumaric acid (serial number: HA062209, purity ≥ 98%), ferulic acid (serial number: 1135-24-6, purity ≥ 98%), ellagic acid (serial number: HE230830, purity ≥ 98%), galloylpaeoniflorin (serial number: HG003027, purity ≥ 98%), luteoloside (serial number: HL090354, purity ≥ 98%), 1,2,3,4,6-penta-O-galloylglucose (serial number: HA060604, purity ≥ 98%), diosmin (serial number: HD304950, purity ≥ 98%), neodiosmin (serial number: HN304948, purity ≥ 98%), resveratrol (serial number: HR047253, purity ≥ 98%), mudanpioside C (serial number: HM185102, purity ≥ 98%), benzoyloxypaeoniflorin (serial number: HA062916, purity ≥ 98%), benzoylpaeoniflorin (serial number: HB003022, purity ≥ 98%), and paeonol (serial number: HP183509, purity ≥ 98%), were purchased from Baoji Chenguang Biotechnology Co., Ltd. (Baoji, China). 1,2,3,6-tri-O-galloylglucose (serial number: T877329, purity ≥ 98%) was purchased from Shanghai Maclin Biochemical Technology Co., Ltd. (Shanghai, China). A Shimadzu Nexera XR high-performance liquid chromatography system, an automatic injection system, and a diode array detector were from Japan Shimadzu Corporation (Tokyo, Japan). A ShimPack Scepter C_18_ chromatographic column (4.6 mm × 250 mm, 5 μm, Shimadzu Experimental Equipment Co., Ltd., Shanghai, China) was used in the study. An AKQ-300DA desktop numerical control ultrasonic cleaner (Kunshan Ultrasonic Instrument Co., Ltd., Kunshan, China), an electronic analytical balance (AUW220D type, Shimadzu Enterprise Management Co., Ltd., Shanghai, China), a pure water/supersaturated water integrated system (Direct-Q^®^5, Merck Millipore, Darmstadt, Germany), a rapid micro-volume freezing centrifuge (D3024R, Beijing Dalong Xingchuang Experimental Instruments Co., Ltd., Beijing, China), a vortex shaker (MX-F, Wuhan Saiwei Biotechnology Co., Ltd., Wuhan, China), an ultrasonic cleaner (JP-040S, Shenzhen Jiemeng Cleaning Equipment Co., Ltd., Shenzhen, China), pipettes (2.0–20.0 μL, 20.0–200 μL, 200–1000 μL, Eppendorf, Hamburg, Germany), a chromatography system (UltiMate 3000 RS, Thermo Fisher Scientific, Waltham, MA, USA), and a mass spectrometer (Q Exactive high-resolution mass spectrometer, Thermo Fisher Scientific, Waltham, MA, USA) were used in the study.

The EPRP was obtained from Moutan Cortex, and the Moutan Cortex medicinal material sample was authenticated as genuine by Professor Siwang Wang from the Northwest University of Traditional Chinese Medicine. Specific information can be found in Table 5. The medicinal materials were provided by Shaanxi Fengdan Zhengyuan Biotechnology Co., Ltd.,China., as shown in Table 5.

### 3.2. Experimental Methods

#### 3.2.1. Solution Preparation

##### Preparation of the EPRP Sample Solution

Approximately 1 g of EPRP (sieved through a no. 4 sieve) was precisely weighed and placed in a stoppered conical flask. Then, 100 mL of 70% MeOH (*v*/*v*) in water was accurately added, the flask was closed tightly, and the weight was recorded. The mixture was subjected to ultrasound treatment for 45 min (power: 250 W, frequency: 40 kHz) and then allowed to cool. The weight was recorded again, and 70% MeOH (*v*/*v*) in water was used to make up for the loss in weight. The mixture was mixed well and centrifuged at 4000 rpm for 10 min. The supernatant was collected and filtered through a 0.22-μm micropore filter membrane to obtain the solution.

##### Preparation of the Reference Standard Solution

Appropriate amounts of 1-galloylglucose, gallic acid, methyl gallate, oxypaeoniflora, paeonolide, apiopaeonoside, albiflorin, paeoniflorin, p-coumaric acid, ferulic acid, 1,2,3,6-tetragalloylglucose, ellagic acid, galloylpaeoniflorin, luteoloside, 1,2,3,4,6-O-penta-galloylglucose, diosmin, neodiosmin, resveratrol, mudanpioside C, benzoyloxypaeoniflorin, benzoylpaeoniflorin, and paeonol were weighed and mixed with methanol to achieve a concentration of 505.19, 200.41,120.15, 335.16, 109.96, 180.12, 2559.76, 4919.60, 205.02, 230.30, 539.98, 159.94, 539.49, 259.70, 2091.32, 2015.20, 45.08, 285.18, 510.09, 415.03, 380.24, and 1070.16 μg·mL^−1^, respectively. These were used as the reserve solutions.

#### 3.2.2. HPLC EPRP Fingerprint Analysis and Content Determination of the 22 Components

The HPLC chromatographic column was a ShimPack Scepter C_18_ column (4.6 mm × 250 mm, 5 μm, Shimadzu Experimental Equipment Co., Ltd., Shanghai, China). Mobile phase A was acetonitrile, and mobile phase B was a 0.1% formic acid aqueous solution. These were used in a gradient elution shown in Supplementary Table S1. The detection wavelength was 270 nm, the flow rate was 1.0 mL·min^−1^, the column temperature was 30 °C, and the injection volume was 10 μL.

#### 3.2.3. HPLC-MS/MS Feature Peak Identification

The HPLC-MS/MS conditions were as follows: ion source, electrospray ionization source; ESI scanning method, positive and negative ion switching scanning; detection method, full mass/dd-MS2; resolution: 70,000 full mass, 17,500 dd-MS2; scan range, 100.0–1500.0 m/z; electrospray voltage, 3.2 kV (positive and negative); capillary temperature, 300; collision gas: high-purity argon (purity ≥ 99.999%); collision energy (N), 30, 40, and 60; sheath gas: nitrogen (purity ≥ 99.999%), 40 arb; auxiliary gas, nitrogen (purity ≥ 99.999%), 15 arb, 350 °C; and data acquisition time, 110.0 min.

#### 3.2.4. Data Analysis

An evaluation of the chromatographic fingerprints was performed using chemometrics methods, incorporating results from the Similarity Evaluation System for Chromatographic Fingerprint of Traditional Chinese Medicine software (version 2012; National Pharmacopoeia Committee, Beijing, China), CA (using SPSS, version 23.0; IBM, Armonk, NY, USA), PCA, and PLS-DA (using SIMCA, version 14.1; Sartorius Stedim Data Analytics AB, Umeå, Sweden).

## 4. Conclusions

HPLC was used to create fingerprints of EPRP samples from different regions. High-resolution mass spectrometry was used to identify the fingerprint peaks; for the first time, the compound [(1-methoxy-1-oxononan-3-yl) 3-hydroxynonanoate] was identified in EPRP samples. Similarity and stoichiometric analyses (CA, PCA, and PLS-DA) were used to analyze the fingerprints of the 13 EPRP batches. From the 30 common peaks, 22 chemical markers were screened and quantitatively determined. The markers, guided by component structure theory, identified the unique component characteristics of EPRP from different regions and provided a valuable reference for the quality evaluation of this herbal medicine. Given the efficacy and comprehensiveness of the HPLC fingerprinting method in combination with chemometrics, as demonstrated in this study, we further advise that this approach provides a valuable reference for assessing the authenticity, quality, and development of herbal medicines or other related food and pharmaceutical products.

## Figures and Tables

**Table 1:** Analytical method validation results of fingerprint analysis.

Peak No.	RSD of Relative Retention Time (%)			RSD of Relative Peak Area (%)		
	Precision (*n* = 6)	Reproducibility (*n* = 6)	Stability (*n* = 6)	Precision (*n* = 6)	Reproducibility (*n* = 6)	Stability (*n* = 6)
1	0.2	0.2	0.1	1.2	1.5	1.3
2	0.2	0.1	0.1	2.2	2.8	2.1
3	0.2	0.1	0.1	1.8	2.8	1.2
4	0.1	0.2	0.1	2.0	1.7	2.2
5	0.2	0.1	0.2	1.4	2.6	3.2
6	0.2	0.2	0.2	1.6	1.3	2.3
7	0.2	0.3	0.1	1.2	2.5	2.8
8	0.2	0.2	0.1	1.8	2.9	2.2
9	0.3	0.3	0.1	2.0	3.1	1.9
10	0.1	0.1	0.2	2.0	2.0	2.4
11	0.2	0.3	0.1	1.4	1.8	2.1
12	0.2	0.2	0.2	2.5	2.7	2.4
13	0.2	0.2	0.2	2.1	2.2	3.0
14	0.1	0.2	0.2	2.1	2.8	2.6
15	0.1	0.2	0.2	2.2	1.4	2.2
16	0.2	0.1	0.1	2.0	2.2	2.2
17	0.2	0.3	0.2	2.6	2.4	2.1
18	0.2	0.3	0.3	2.9	1.9	2.3
19	0.2	0.1	0.1	1.9	1.7	2.5
20	0.1	0.2	0.3	2.4	3.8	1.7
21	0.2	0.1	0.1	2.2	1.9	3.0
22	0.2	0.1	0.2	2.2	2.4	2.0
23	0.2	0.1	0.3	3.1	3.2	3.1
24	0.2	0.1	0.1	2.1	3.5	1.8
25	0.2	0.1	0.1	2.8	1.9	3.0
26	0.2	0.2	0.1	2.0	2.6	2.0
27	0.2	0.1	0.1	2.9	3.7	2.8
28	0.1	0.1	0.2	2.7	2.1	2.6
29	0.0	0.0	0.0	0.0	0.0	0.0
30	0.2	0.2	0.2	1.8	2.1	1.9

RSD: relative standard deviation.

**Table 2:** Characterization of the 13 EPRPs components by LC–MS/MS.

Peak	RT (min)	Formula	Calculated (*m*/*z*)	Experimental (*m*/*z*)	Error (ppm, −10–+10%)	Ion Mode	Parent and Major Fragment Ions	Identification	Reference
1	2.48	C_12_H_22_O_11_	341.10893	341.10843	−1.48	ESI(−)	341.10843, 179.05504, 119.03349	Disaccharide	[19]
	3.57	C_20_H_18_O_14_	481.06238	481.06281	0.90	ESI(−)	481.06281, 463.10895, 300.92661	Hexahydroxydiphenoyl-β-D-glucose	[19]
2	4.32	C_13_H_16_O_10_	331.06707	331.06702	3.15	ESI(−)	331.06702, 69.01402, 125.02312, 119.03355, 97.02768	1-Galloylglucose	[19]
3	5.83	C_7_H_6_O_5_	169.01425	169.01323	0.47	ESI(−)	169.01323, 125.02302, 107.01244, 97.02798, 81.03304, 79.01734	Gallic acid	[2,19,22,23,24]
4	18.04	C_8_H_8_O_5_	183.0299	183.02902,	1.22	ESI(−)	183.02902, 153.01816, 123.94514, 112.98424	Methyl gallate	[2,19,22,23,24]
5	18.09	C_23_H_28_O_12_	495.1508	495.15063	1.88	ESI(−)	495.15063, 477.13913, 461.10362, 432.88199, 333.09741, 174.95514, 121.02813	Oxypaeoniflora	[2,19,22,23,24]
6	24.19	C_20_H_28_O_12_	461.16535	461.16495	−0.88	ESI(+)	461.16495, 329.12238, 167.07016, 166.06464, 152.04645, 121.06516, 91.05473	Paeonolide	[2,19]
7	27.07	C_20_H_28_O_12_	461.16535	461.16486	−1.08	ESI(+)	461.16486, 329.12262, 167.07013, 166.06442, 152.04657, 121.06499, 91.05473	Apiopaeonoside	[2,19]
8	27.52	C_23_H_28_O_11_	481.17044	481.17026	−0.38	ESI(+)	481.17026, 449.06903, 319.11795, 301.106693, 121.02854, 91.05478	Albiflorin	[2,19]
9	31.09	C_23_H_28_O_11_	479.15588	479.15558	−0.64	ESI(−)	479.15558, 317.04114, 299.09921, 121.02808	Mudanpioside A	[19,22]
10	32.20	C_23_H_28_O_11_	481.17044	481.17014	−0.63	ESI(+)	481.17014, 463.15942, 305.10175, 301.10657, 121.02882, 91.05466	Paeoniflorin	[2,19,25,26]
				525.16138	0.02	ESI(−)	525.16138, 163.03908, 145.03143		
11	33.82	C_9_H_8_O_3_	163.04007	163.03912	−3.78	ESI(−)	163.03912, 119.04891	*p*-Coumaric acid	[2,19]
			165.05462	165.05438	−4.89	ESI(+)	165.05438, 121.02860, 119.04945		
12	36.76	C_27_H_32_O_16_	613.17631	613.17596	−0.57	ESI(+)	613.17596, 465.12424, 451.12222, 121.02844	Suffruticoside A	[2,16,19,22]
13	40.46	C_10_H_10_O_4_	193.05063	193.04996	−0.67	ESI(−)	193.04994, 177.01848, 149.02336, 134.03482, 121.02821	Ferulic acid	[2,19]
14	41.33	C_34_H_28_O_22_	787.09995	787.09998	0.81	ESI(−)	787.09998, 635.08624, 483.07483, 465.06735, 313.05634, 169.01311, 125.02303	1,2,3,6-Tetragalloylglucose	[2,19]
15	43.04	C_14_H_6_O_8_	300.99899	300.99869	−1.00	ESI(−)	300.99869, 229.01344, 213.01730, 185.02344, 145.02818	Ellagic acid	[2,19,22,24]
16	44.81	C_30_H_32_O_15_	631.16684	631.16681	0.05	ESI(−)	631.16681, 613.21515, 463.21848, 169.01399, 125.02306	Galloylpaeoniflorin	[2,16,19,22]
17	46.23	C_21_H_20_O_11_	447.09328	447.09396	3.97	ESI(−)	447.09296, 285.03903, 151.00262, 137.02318, 107.01238	Luteoloside	[2,19,22,23,24]
18	52.68	C_41_H_32_O_26_	939.11090	939.11139	1.68	ESI(−)	939.11139, 245.01207, 205.01122, 165.01825, 137.02319	1,2,3,4,6-O-Penta-galloylglucose	[2,19,22]
19	55.26	C_30_H_32_O_14_	615.17193	615.17169	−0.39	ESI(−)	615.17169, 477.14008, 453.12745, 283.07870, 151.03888	Mudanpioside H	[26,27]
20	63.05	C_48_H_36_O_30_	1091.12186	1091.12231	0.42	ESI(−)	1091.12231, 939.10919, 769.08301, 617.45886, 447.12881, 169.01427, 125.02311	Hexagallylglucose	[26]
21	67.06	C_28_H_32_O_15_	607.16684	607.16656	−0.46	ESI(−)	607.16656, 299.05560, 151.00238	Diosmin	[26]
22	67.76	C_28_H_32_O_15_	607.16684	607.16687	0.04	ESI(−)	607.16687, 461.10913, 299.05588, 151.00250, 121.02819	Neodiosmin	[19]
23	69.59	C_14_H_12_O_3_	227.07137	227.07635	4.99	ESI(−)	227.07635, 185.06354, 143.04523, 117.03289, 93.03291	Resveratrol	[19]
24	82.26	C_30_H_32_O_13_	599.17701	599.17712	0.18	ESI(−)	599.17712, 447.12955, 429.11731, 165.01834, 121.02819, 477.14102	Mudanpioside C	[2,19,22,27]
25	87.64	C_30_H_32_O_13_	599.17701	599.17645	−0.94	ESI(−)	599.17645, 447.13055, 429.12125, 165.01820, 121.02808, 461.14532	Benzoyloxypaeoniflorin	[19,22,27]
26	92.96	C_30_H_32_O_12_	583.1821	583.18115	−1.62	ESI(−)	583.18115, 461.14685, 327.14453, 165.01822, 121.02804, 431.13568	Benzoylpaeoniflorin	[19,22,27]
27	93.48	C_17_H_10_O_5_	269.04555	269.04556	0.06	ESI(−)	269.04556, 241.04974, 225.05550, 251.03377, 151.03888, 179.03412	Apigenin	[19,22,23,24,28]
28	94.92	C_17_H_10_O_7_	301.03538	301.03415	−0.44	ESI(−)	301.03415, 273.0766, 283.03781, 257.04898, 151.03893, 179.03413, 137.02318	Quercetin	[23,24,29]
29	96.01	C_9_H_10_O_3_	165.05572	165.05466	0.22	ESI(−)	165.05466, 151.03893, 121.02816	Paeonol	[2,8,19,22]
30	104.11	C_19_H_36_O_5_	345.26355	345.26187	−4.87	ESI(+)	345.26187, 101.07131, 174.07482, 145.05495	(1-Methoxy-1-oxononan-3-yl) 3-hydroxynonanoate	

**Table 3:** Linear regression equations, precision, stability, repeatability, and recovery of the 22 compounds in EPRP.

No.	Regression Equation	R^2^	Linear Range (µg/mL)	Precision (RSD, %)		Stability (RSD, %; *n* = 6)	Repeatability (RSD, %; *n* = 6)	Recovery	
				Intraday (*n* = 6)	Interday (*n* = 6)			Mean	RSD, %
Peak 2	Y = 7737.76X − 9933.61	0.9998	1.01–505.19	0.6	0.7	0.8	1.8	97.1	1.2
Peak 3	Y = 25,723.30X + 3222.93	0.9997	0.40–200.41	1.0	1.0	0.8	2.1	98.3	2.0
Peak 4	Y = 37,745.24X + 17,681.39	0.9994	0.24–120.15	0.9	1.1	0.9	0.9	99.5	1.3
Peak 5	Y = 134,21.70X − 20,453.71	0.9995	0.67–335.16	1.6	1.6	1.1	1.8	100.2	1.6
Peak 6	Y = 13,636.79X − 1450.19	0.9996	0.22–109.96	1.2	1.1	1.2	1.0	100.0	1.1
Peak 7	Y = 15,990.49X + 299.01	0.9998	0.36–180.12	1.4	1.5	0.7	1.5	99.6	2.0
Peak 8	Y = 771.42X − 3812.41	0.9999	5.12–2559.76	1.1	1.2	1.1	1.4	98.6	2.3
Peak 10	Y = 1019.18X − 20,443.37	0.9996	9.84–4919.60	1.5	1.3	0.6	0.8	97.9	1.3
Peak 11	Y = 25,971.99X + 13,588.29	0.9997	0.41–205.02	1.3	1.4	0.9	2.3	98.9	1.7
Peak 13	Y = 21,999.70X + 26,584.48	0.9993	0.46–230.30	1.8	1.9	1.0	1.6	97.8	2.4
Peak 14	Y = 19,932.13X − 17,677.85	0.9999	1.08–539.98	1.4	1.4	1.0	1.5	97.0	1.9
Peak 15	Y = 36,004.36X + 23,224.70	0.9997	0.32–159.94	1.2	1.1	1.2	0.8	100.6	1.7
Peak 16	Y = 8615.43X − 20,450.92	0.9996	1.08–539.49	0.5	0.5	0.7	1.9	97.1	1.2
Peak 17	Y = 19,569.43X − 14,724.95	0.9998	0.52–259.70	1.1	1.3	0.9	1.5	98.5	1.6
Peak 18	Y = 19,647.44X − 16,6391.15	0.9998	4.18–2091.32	0.9	1.1	0.7	0.8	98.8	1.8
Peak 21	Y = 27,825.61X + 54,073.20	0.9998	4.03–2015.20	1.5	1.7	1.3	1.6	100.5	2.0
Peak 22	Y = 86,284.91X − 24,764.69	0.9996	0.09–45.08	1.1	1.1	1.2	1.0	99.4	1.9
Peak 23	Y = 35,971.65X − 17,678.40	0.9999	0.57–285.18	1.3	1.4	0.9	2.1	99.8	1.6
Peak 24	Y = 10,088.36X + 20,451.43	0.9996	1.02–510.09	1.5	1.5	1.2	1.5	98.3	1.2
Peak 25	Y = 13,377.50X + 13,611.96	0.9996	0.83–415.03	1.8	1.6	1.3	0.6	99.4	2.2
Peak 26	Y = 13,380.92X + 1614.99	0.9997	0.76–380.24	1.6	1.7	1.9	1.7	97.9	1.8
Peak 29	Y = 42,258.37X + 204,513.93	0.9999	2.14–1070.16	1.7	1.9	0.9	1.3	97.4	1.4

**Table 4:** Contents of the 22 components of EPRP across 13 batches (n = 3, mean ± SD, mg·g^−1^).

Sample No.	Terpene Nucleoside Composition							Phenolic and Phenolic Glycoside Composition					
	Peak 5	Peak 8	Peak 10	Peak 16	Peak 24	Peak 25	Peak 26	Peak 6	Peak 7	Peak 11	Peak 13	Peak 23	Peak 29
S1	1.6599 ± 0.0108	5.3649 ± 0.0601	7.3341 ± 0.0792	4.9352 ± 0.0701	1.0578 ± 0.0097	0.8981 ± 0.0079	1.2841 ± 0.0094	2.1125 ± 0.0207	4.7117 ± 0.0476	0.1352 ± 0.0008	0.4228 ± 0.0023	0.7512 ± 0.0074	41.2066 ± 0.2637
S2	1.6162 ± 0.0091	4.5757 ± 0.0421	7.4419 ± 0.0640	5.0300 ± 0.0407	1.0387 ± 0.0102	0.9133 ± 0.0059	1.1968 ± 0.0165	2.1068 ± 0.0158	4.6924 ± 0.0507	0.1223 ± 0.0009	0.4292 ± 0.0029	0.7420 ± 0.0082	39.7026 ± 0.3851
S3	1.6470 ± 0.0175	6.4132 ± 0.0635	7.6502 ± 0.0551	4.7689 ± 0.0362	1.0492 ± 0.0090	0.8590 ± 0.0066	1.1544 ± 0.0113	2.1000 ± 0.0200	4.7207 ± 0.0633	0.1370 ± 0.0009	0.4228 ± 0.0027	0.7099 ± 0.0073	42.6900 ± 0.4440
Mean	1.64	5.45	7.48	4.91	1.05	0.89	1.21	2.11	4.71	0.13	0.42	0.73	41.20
Median	1.64	5.36	7.44	4.94	1.05	0.90	1.20	2.11	4.71	0.14	0.42	0.74	41.21
S4	0.9767 ± 0.0108	0.4453 ± 0.0043	14.8552 ± 0.1278	6.1779 ± 0.0513	0.7435 ± 0.0059	3.6834 ± 0.0306	2.5412 ± 0.0213	0.1346 ± 0.0017	1.2100 ± 0.0119	0.0379 ± 0.0003	0.2631 ± 0.0020	0.4271 ± 0.0045	38.1037 ± 0.2743
S5	0.9098 ± 0.0077	0.4517 ± 0.0057	15.6004 ± 0.1186	6.0402 ± 0.0562	0.7071 ± 0.0063	3.2292 ± 0.0229	3.1901 ± 0.0316	0.1426 ± 0.0011	1.1558 ± 0.0124	0.0324 ± 0.0003	0.2567 ± 0.0026	0.4896 ± 0.0066	37.1354 ± 0.4011
S10	0.7043 ± 0.0073	2.0333 ± 0.0268	6.5314 ± 0.0666	14.7449 ± 0.1357	0.6541 ± 0.0048	8.6427 ± 0.0856	7.0502 ± 0.1001	0.5535 ± 0.0047	0.9635 ± 0.0095	0.0475 ± 0.0005	0.2970 ± 0.0022	0.2553 ± 0.0019	39.4265 ± 0.3430
S11	0.6950 ± 0.0066	1.9566 ± 0.0205	7.0459 ± 0.0627	14.1734 ± 0.1389	0.5220 ± 0.0045	8.4212 ± 0.0699	7.1070 ± 0.0760	0.5512 ± 0.0053	0.9574 ± 0.0108	0.0450 ± 0.0004	0.3124 ± 0.0025	0.2625 ± 0.0027	40.1723 ± 0.3616
Mean	0.82	1.22	11.01	10.28	0.66	5.99	4.97	0.35	1.07	0.04	0.28	0.36	38.71
Median	0.81	1.20	10.95	10.18	0.68	6.05	5.12	0.35	1.06	0.04	0.28	0.34	38.77
S6	0.6724 ± 0.0046	1.2447 ± 0.0122	12.1519 ± 0.0911	16.8098 ± 0.2538	0.4999 ± 0.0043	4.8876 ± 0.0386	8.4306 ± 0.0708	0.5393 ± 0.0035	0.7953 ± 0.0075	0.0475 ± 0.0004	0.2543 ± 0.0022	0.1946 ± 0.0015	31.0533 ± 0.295
S7	0.6359 ± 0.0061	1.1948 ± 0.0102	11.7610 ± 0.1211	16.2274 ± 0.1412	0.4861 ± 0.0037	4.7827 ± 0.0435	8.4314 ± 0.1147	0.5119 ± 0.0057	0.7972 ± 0.0128	0.0484 ± 0.0005	0.2544 ± 0.0023	0.1931 ± 0.0017	30.5423 ± 0.2474
S8	0.7036 ± 0.0085	0.7597 ± 0.0081	7.6993 ± 0.0731	3.9565 ± 0.0285	0.5627 ± 0.0053	2.1377 ± 0.0207	2.1084 ± 0.0228	0.9282 ± 0.0123	1.2095 ± 0.0132	0.0407 ± 0.0003	0.1920 ± 0.0020	0.1979 ± 0.0022	27.732 ± 0.2745
S9	0.8402 ± 0.0118	0.7178 ± 0.0073	9.1346 ± 0.0804	3.6575 ± 0.0358	0.5899 ± 0.0052	1.6759 ± 0.0136	2.5412 ± 0.0262	0.1179 ± 0.0011	0.1348 ± 0.0015	0.0379 ± 0.0003	0.1707 ± 0.0014	0.2187 ± 0.0023	28.2265 ± 0.2540
S12	0.9767 ± 0.0074	0.4453 ± 0.0048	15.0158 ± 0.1937	3.2168 ± 0.0273	0.7539 ± 0.0049	3.9019 ± 0.0496	2.6210 ± 0.0372	0.1489 ± 0.0013	0.1211 ± 0.0011	0.0393 ± 0.0004	0.2672 ± 0.0021	0.4269 ± 0.0037	28.2636 ± 0.2007
S13	1.0487 ± 0.0099	0.5617 ± 0.0059	16.3125 ± 0.1501	3.0643 ± 0.0297	0.7435 ± 0.0063	3.6834 ± 0.0320	2.5412 ± 0.0280	0.1346 ± 0.0013	0.1239 ± 0.0014	0.0414 ± 0.0003	0.2773 ± 0.0022	0.4687 ± 0.0047	28.7210 ± 0.2527
Mean	0.81	0.82	12.01	7.82	0.61	3.51	4.45	0.40	0.53	0.04	0.24	0.28	29.09
Median	0.77	0.74	11.96	3.81	0.58	3.79	2.58	0.33	0.47	0.04	0.25	0.21	28.47
Sample No.	Tannic Acid Composition							Flavonoid Composition					
	Peak 2	Peak 3	Peak 4	Peak 14	Peak 15			Peak 18	Peak 17	Peak 21	Peak 22		
S1	0.1299 ± 0.0011	0.2615 ± 0.0017	1.0725 ± 0.0077	1.4782 ± 0.0112	2.4600 ± 0.0234			5.7980 ± 0.0499	0.0971 ± 0.0018	2.1182 ± 0.0265	0.0368 ± 0.0004		
S2	0.1152 ± 0.0007	0.2639 ± 0.0025	1.0027 ± 0.0084	1.4590 ± 0.0128	2.4612 ± 0.0236			5.7290 ± 0.0533	0.0831 ± 0.0010	2.0606 ± 0.0202	0.0358 ± 0.0005		
S3	0.1310 ± 0.0010	0.2626 ± 0.0023	1.0414 ± 0.0126	1.5885 ± 0.0129	2.8964 ± 0.0217			5.6948 ± 0.0421	0.0910 ± 0.0008	2.2025 ± 0.0227	0.0368 ± 0.0003		
Mean	0.13	0.26	1.04	1.51	2.61			5.74	0.09	2.13	0.04		
Median	0.13	0.26	1.04	1.48	2.46			5.73	0.09	2.12	0.04		
S4	0.0831 ± 0.0008	0.2473 ± 0.0014	0.3811 ± 0.0046	13.6179 ± 0.1158	0.8831 ± 0.0080			4.1548 ± 0.0453	0.0283 ± 0.0003	1.4791 ± 0.0109	0.0322 ± 0.0003		
S5	0.0835 ± 0.0011	0.2702 ± 0.0029	0.4196 ± 0.0041	14.6995 ± 0.1132	0.9072 ± 0.0062			4.0122 ± 0.0530	0.0274 ± 0.0002	1.6549 ± 0.0161	0.0305 ± 0.0002		
S10	0.0198 ± 0.0002	0.0390 ± 0.0004	6.8615 ± 0.0604	15.6645 ± 0.1128	8.8575 ± 0.0779			2.5471 ± 0.0222	0.0410 ± 0.0005	2.2953 ± 0.0213	0.0129 ± 0.0001		
S11	0.0510 ± 0.0005	0.0320 ± 0.0003	6.9407 ± 0.0639	14.1146 ± 0.1172	8.9249 ± 0.0821			2.5088 ± 0.0233	0.0142 ± 0.0001	2.2800 ± 0.0210	0.0127 ± 0.0001		
Mean	0.06	0.15	3.65	14.52	4.89			3.31	0.03	1.93	0.02		
Median	0.05	0.14	3.64	14.41	4.88			3.28	0.03	1.97	0.02		
S6	0.0198 ± 0.0002	0.0386 ± 0.0004	6.4110 ± 0.0462	13.3113 ± 0.0945	6.2703 ± 0.0828			2.3041 ± 0.0127	0.0410 ± 0.0003	2.2953 ± 0.0145	0.0211 ± 0.0002		
S7	0.0199 ± 0.0001	0.0384 ± 0.0003	6.3989 ± 0.0499	13.4623 ± 0.1131	6.2415 ± 0.0593			2.3070 ± 0.0247	0.0389 ± 0.0003	2.2953 ± 0.0250	0.0215 ± 0.0002		
S8	0.0660 ± 0.0007	0.4994 ± 0.0043	0.8628 ± 0.0084	10.3181 ± 0.1156	0.2293 ± 0.0019			1.7324 ± 0.0158	0.0283 ± 0.0002	0.6344 ± 0.0043	0.0147 ± 0.0001		
S9	0.0693 ± 0.0006	0.4651 ± 0.0040	0.7146 ± 0.0066	11.4209 ± 0.0959	0.2192 ± 0.0020			1.6356 ± 0.0152	0.0271 ± 0.0003	0.6348 ± 0.0058	0.0182 ± 0.0002		
S12	0.1003 ± 0.0010	0.2479 ± 0.0017	0.4050 ± 0.0038	13.6039 ± 0.1483	0.8804 ± 0.0100			4.1983 ± 0.0474	0.0283 ± 0.0002	1.5490 ± 0.0260	0.0327 ± 0.0003		
S13	0.0929 ± 0.0009	0.2703 ± 0.0023	0.3807 ± 0.0035	11.4209 ± 0.0971	0.7961 ± 0.0075			4.1548 ± 0.0391	0.0271 ± 0.0003	1.7321 ± 0.0170	0.0349 ± 0.0003		
Mean	0.06	0.26	2.53	12.26	2.44			2.72	0.03	1.52	0.02		
Median	0.02	0.26	0.79	12.37	0.84			2.30	0.03	1.64	0.02		

**Table 5:** EPRP sample information.

No.	Collection Date	Place of Origin
S1	17 April 2025	Heyang, Shaanxi
S2	17 April 2025	Heyang, Shaanxi
S3	17 April 2025	Heyang, Shaanxi
S4	19 April 2025	Chengdu, Sichuan
S5	19 April 2025	Chengdu, Sichuan
S6	21 April 2025	Tongling, Anhui
S7	21 April 2025	Tongling, Anhui
S8	22 April 2025	Heze, Shandong
S9	22 April 2025	Heze, Shandong
S10	23 April 2025	Dianjiang, Chongqing
S11	23 April 2025	Dianjiang, Chongqing
S12	20 April 2025	Luoyang, Henan
S13	20 April 2025	Luoyang, Henan

## Data Availability

All data are contained within the article and its Supplementary Materials.

## Supplementary Materials

The following supporting information can be downloaded at: https://www.mdpi.com/article/10.3390/molecules31040588/s1. Table S1: Gradient elution program.

## Abbreviations

The following abbreviations are used in this manuscript:

RSD	Relative standard deviation
HPLC-DAD-ESI-MS/MS	High-performance liquid chromatography–diode array detector–electrospray ionization–mass spectrometry/mass spectrometry
EPRP	Epidermis of the root bark of peony
CA	Cluster analysis
PCA	Principal component analysis
PLS-DA	Partial least squares–discriminant analysis
TCM	Traditional Chinese medicine
