# Supplementary material for: Quality Evaluation of the Root Bark Epidermis of Peony by HPLC-DAD-ESI-MS/MS
Source: Molecules. 2026 Feb 8;31(4):588. doi: 10.3390/molecules31040588 (PMC12942668; doi:10.3390/molecules31040588)
Supplement: Supplementary file 1 [file molecules-31-00588-s001.zip › molecules-4013959-supplementary.pdf]

Supplementary materials

Supplementary Table S1. Gradient elution program

Time (min)	Mobile phase A (%)	Mobile phase B (%)
0 – 10	7 → 10	93 → 90
10 – 35	10 → 17	90 → 83
35 – 80	17 → 21	83 → 79
80 – 90	21 → 40	79 → 60
90 – 100	40 → 55	60 → 45
100 – 110	55 → 45	45 → 55
